# Factors associated with health-seeking patterns among internally displaced persons in complex humanitarian emergency, Northeast Nigeria: a cross-sectional study

**DOI:** 10.1186/s13031-023-00552-7

**Published:** 2023-11-08

**Authors:** Saheed Gidado, Melton Musa, Ahmed Ibrahim Ba’aba, Lilian Akudo Okeke, Patrick M Nguku, Idris Suleman Hadejia, Isa Ali Hassan, Ibrahim Muhammad Bande, Martins Onuoha, Gideon Ugbenyo, Ntadom Godwin, Rabi Usman, Jibrin Idris Manu, Abede Momoh Mohammed, Muhammad Maijawa Abdullahi, Mohammed Isa Bammami, Pekka Nuorti, Salla Atkins

**Affiliations:** 1 Health Sciences Unit, Faculty of Social Sciences, Tampere University, Tampere, Finland; 2 African Field Epidemiology Network, Borno State Field Office, Maiduguri, Nigeria; 3 African Field Epidemiology Network, Yobe State Field Office, Damaturu, Nigeria; 4 African Field Epidemiology Network, Adamawa State Field Office, Yola, Nigeria; 5 African Field Epidemiology Network, Country Office, Abuja, Nigeria; 6 Department of Community Medicine, Ahmadu Bello University, Zaria, Kaduna State Nigeria; 7 Borno State Ministry of Health, Maiduguri, Borno State Nigeria; 8 Department of Disease Control and Immunization, Yobe State Primary Health Care Board, Damaturu, Yobe State Nigeria; 9 Nigerian Correctional Service, Adamawa State Office, Yola, Adamawa State Nigeria; 10 Nigeria Field Epidemiology and Laboratory Training Program, Abuja, Nigeria; 11 Epidemiology Division, Federal Ministry of Health, Abuja, Nigeria; 12 Resolve to Save Lives, Abuja, Nigeria; 13 World Health Organization, Borno State Office, Maiduguri, Nigeria; 14 Yobe State Hospitals Management Board, Damaturu, Yobe State Nigeria

**Keywords:** Health-seeking, Internally displaced persons, Complex humanitarian emergency, Nigeria, Cross-sectional study

## Abstract

**Background:**

Currently, over two million persons are internally displaced because of the complex humanitarian emergency in Nigeria’s northeast region. Due to crowded and unsanitary living conditions, the risk of communicable disease transmission, morbidity, and mortality among this population is high. This study explored patterns and factors associated with health-seeking among internally displaced persons (IDPs) in northeast Nigeria to inform and strengthen disease surveillance and response activities.

**Methods:**

In a cross-sectional study conducted during June–October 2022, we employed stratified sampling technique to select 2,373 IDPs from 12 IDPs camps. A semi-structured tool was used to collect data on health-seeking patterns, socio-demographics, households, and IDPs camps characteristics. We classified health-seeking patterns into three outcome categories: ‘facility care’ (reference category), ‘non-facility care’ (patent medicine vendors, chemists, traditional healers, religious centers), and ‘home care/no care’. We performed complex survey data analysis and obtained weighted statistical estimates. Univariate analysis was conducted to describe respondents’ characteristics and health-seeking patterns. We fitted weighted multivariable multinomial logistic regression models to identify factors associated with health-seeking patterns.

**Results:**

Of 2,373 respondents, 71.8% were 18 to 39 years old, 78.1% were females, and 81.0% had no formal education. Among the respondents, 75.7% (95% CI: 72.9–78.6) sought ‘facility care’, 11.1% (95% CI: 9.1–13.1) sought ‘non-facility care’, while 13.2% (95% CI: 10.9–15.4) practiced ‘home care/no care’. Respondents who perceived illness was severe (Adjusted Odds Ratio (AOR) = 0.15, [95% CI: 0.08–0.30]) and resided in officially-recognized camps (AOR = 0.26, [95% CI: 0.17–0.39]) were less likely to seek ‘non-facility care’ compared to ‘facility care’. Similarly, respondents who resided in officially-recognized camps (AOR = 0.58, [95% CI: 0.36–0.92]), and received disease surveillance information (AOR = 0.42, [95% CI: 0.26–0.67) were less likely to practice ‘home care/no care’ rather than seek ‘facility care’.

**Conclusions:**

This population exhibited heterogeneous patterns of health-seeking at facility and non-facility centers. Perception of illness severity and camps’ status were major factors associated with health-seeking. To enhance surveillance, non-facility care providers should be systematically integrated into the surveillance network while ramping up risk communication to shape perception of illness severity, prioritizing unofficial camps.

**Supplementary Information:**

The online version contains supplementary material available at 10.1186/s13031-023-00552-7.

## Background

Internally displaced persons (IDPs) are persons or groups of persons who have been forced or obliged to flee or to leave their homes or places of habitual residence, in particular as a result of or to avoid the effects of armed conflict, situations of generalized violence, violations of human rights or natural or human-made disasters, and who have not crossed an internationally recognized State border [1]. Following their displacements, most IDPs often reside in camps, makeshift structures, and other camp-like settings with minimal or no basic amenities. These settings are usually characterized by population congestion, overcrowded shelters, poor water, sanitation, and hygiene (WASH), and reduced food security [2]. Expectedly, these conditions heighten the risk of transmission of communicable diseases and other conditions, with high excess morbidity and mortality [3]. In addition, non-vaccination or incomplete vaccination of eligible children against childhood diseases facilitates outbreaks of vaccine-preventable diseases in these settings [4]. Moreover, the presence of IDPs in host communities strains local health systems and negatively impacts health outcomes [5].

In Northeast Nigeria, an armed rebellion by Non-State Armed Groups, which started in 2009, triggered a complex humanitarian emergency (CHE) in this region [6]. Three states in the region, namely Borno, Adamawa, and Yobe (known as BAY States), have witnessed immense armed conflicts and generalized violence with extensive destruction of lives and properties [6]. Since the beginning of the humanitarian crisis over a decade ago, more than two million persons, mostly women and children, have been displaced in the region [7]. Predictably, this CHE has rendered health systems fragile, impacted healthcare delivery services, and undermined disease surveillance and response. This is exemplified by the spate of communicable disease outbreaks with substantial morbidity and mortality, particularly among IDPs in the region [8–10]. Sequel to the prevailing public health situation, the World Health Organization (WHO), in 2016, declared a Grade 3 emergency in the region, indicating a substantial public health emergency necessitating major international response [11].

In light of the increased risk of disease transmission in humanitarian emergencies, particularly with the evolving threat of emerging and re-emerging diseases such as COVID-19, it is imperative to enhance disease surveillance and response among IDPs in this context. According to WHO, timely detection and swift response to disease outbreaks among crisis-affected population is a key priority during humanitarian emergencies [12]. Similarly, many authors have demonstrated that an enhanced epidemiological surveillance system that can promptly detect disease conditions is crucial to timely and effective response to disease outbreaks in refugees camps, IDPs camps, and other congested settings [13]. Apart from enabling prompt detection and timely response to disease outbreaks, surveillance system in humanitarian emergencies also helps to identify public health priorities, monitor trends of disease incidence and mortality, and provide information to health and humanitarian organizations for effective programme planning [14].

Effective disease surveillance and response combines elements of both active and passive surveillance with facility-based and non-facility based approaches [15, 16]. However, during humanitarian emergencies, facility-based surveillance may be underperforming or completely disrupted due to destruction of key infrastructure, including health care facilities [17]. To facilitate prompt disease detection and reporting for timely intervention in this context, it is essential to understand the patterns and factors associated with care seeking among crisis-affected population during illness episodes. Generally, health-seeking patterns reflect an interplay of several factors in the context of the prevailing situation [18]. Although, previous research have explored health-seeking patterns among different populations, most were conducted in stable, non-humanitarian situations [19–21]. In this study, we investigated the patterns and factors associated with health-seeking among IDPs in complex emergency setting in Northeast Nigeria. Beside bridging knowledge gaps in published literature, findings from this study will inform targeted interventions to enhance disease surveillance and response and contribute to the reduction of disease morbidities and mortalities among IDPs in this setting.

## Methods

### Study design

This was a cross-sectional study conducted between June and October 2022 among IDPs residing in selected IDPs camps in Borno, Adamawa, and Yobe States (BAY States).

### Study setting

Borno, Adamawa, and Yobe States (BAY States) are situated in Nigeria’s northeast region. These states share international borders with Niger, Chad, and Cameroun. Borno State has a population of 6,629,190, Adamawa: 4,727,312 and Yobe: 3,757,947 [22]. Administratively, Borno, Adamawa and Yobe have 27, 21 and 17 Local Government Areas (LGAs) or districts respectively. Across these three states, there are about 284 IDPs camps and camp-like settings hosting an estimated 195,901 households and 855,020 IDPs [7]. A number of these camps are designated as official (formal) IDPs camps because of the presence of Government authorities and camp management structure, while several others are unofficial (informal). Health care services are provided by facilities within IDPs camps, particularly the official camps. In addition, several other health facilities in the host communities also serve the IDPs.

### Target population, study population and study participants

The study targeted IDPs residing in IDPs camps in Borno, Adamawa, and Yobe States. The study population were IDPs residing in the 12 IDPs camps selected for the study across the three states. We sampled our study participants from among the study population in the selected camps. Study participants were IDPs with the most recent illness onset in selected households across the 12 IDPs camps.

### Sample size determination

We adopted the approach recommended by Lwanga and Lemeshow to determine the sample size for the study [23]. We assumed an anticipated population proportion (proportion of IDPs that sought facility care during illness), previously reported as 65% [24]. With a confidence level of 95% and a margin of error of 5%, we obtained an effective sample size of approximately 350 per state. To account for the selection of study participants via a sampling method other than simple random sampling, we multiplied the effective sample size by the design effect [25, 26]. In the absence of any previously documented design effect in a similar context, we chose a design effect of 2, resulting in an actual sample size of 700 per state. Based on a non-response rate of 10%, we adjusted the actual sample size to 778 per state to obtain a minimum of 2,334 participants across the three states.

### Selection of IDPs camps

For practical and logistic considerations, we selected 12 IDPs camps across the three states – four camps in each state. We used field assessment data from the International Organization for Migration (IOM), Displacement Tracking Matrix (DTM) – Northeast Nigeria Displacement Report Round 40 (March 2022) to select the study camps [7]. Reports of IOM DTM assessments comprehensively document displaced population estimates at household and individual levels. We adopted essential criteria from the displacement report to select the study IDPs camps. The criteria included: (1) population size of the camps (large camps offering a reasonable representation of IDPs from different LGAs and with varying socio-cultural backgrounds were prioritized), (2) camp status (whether official or unofficial), (3) geographical spread of camps across different LGAs, (4) a mixture of camps supported by different organizations, and (5) camps with minimal security risk to the research team. The sample size for each state was distributed equally among the four selected IDPs camps in the respective states.

### Selection of households and respondents

We adopted a stratified random sampling approach to select households and respondents. This method is one of the recommended approaches for selecting study participants in displacement context [27]. We leveraged the polio eradication program’s house-to-house vaccination teams’ microplanning approach to stratify each IDPs camp into four distinct geographical strata [28]. The sample size for each camp was distributed across the strata proportionate to the population size of each stratum. We then employed a simple random sampling approach via computer generated random numbers to select the required number of households from each stratum. We utilized the polio eradication program’s microplanning enumerated household listing data to facilitate the selection of households^1^. In each selected household, the household member with the most recent illness onset since arrival in the camp was selected and interviewed. However, if the household member with the most recent illness onset was less than 18 years old, the caregiver was interviewed instead.

### Data collection

Locally hired, trained interviewers conducted face-to-face interviews with respondents to collect data electronically using open data kit (ODK) – an open-source mobile data collection platform uploaded on internet enabled android device [29]. The interviewers received a 2-day classroom training and a day practical field training. Training content included interview techniques, data instrument and survey items, ODK, security awareness, and adherence to COVID-19 preventive measures among others. The semi-structured data collection instrument was initially developed in English language but translated into local language (Hausa) before data collection. We field-tested the data collection instrument in a camp in Borno State to assess the appropriateness of the survey items and establish the content validity of the data tool.

### Study variables

The outcome variable for this study was health-seeking behavior – defined as any action (or inaction) undertaken by individuals who perceive themselves to have a health problem or to be ill for the purpose of finding an appropriate remedy [18]. For data analysis, we grouped the patterns of health-seeking behavior into three outcome categories: (1) facility care, (2) non-facility care, and (3) ‘home care/no care’. Facility care included seeking healthcare at public, private, faith-based, or Non-Governmental Organizations-supported health facilities offering inpatient and/or outpatient healthcare services. Non-facility care entailed seeking care from patent medicine vendors (PMVs), chemists, traditional healers, and religious centers. Other actions such as “doing nothing at all,” home remedy, and prayers were categorized as ‘home care/no care’. We designated facility care as the reference outcome category and compared each of the other two outcome categories (non-facility care and ‘home care/no care’) with this reference category. The explanatory variables were grouped into different sections. Respondents’ socio-demographic data included age, gender, highest formal education attained, marital status, religion, and occupation. Explanatory variables regarding household characteristics included monthly household income, duration of residence in IDPs camps, household decision-maker on health-seeking, and household distance to the nearest health facility. Data on IDPs camps’ characteristics included camp status (official versus unofficial), year of camp establishment, and perception of IDPs camp security. The section on most recent illness captured data on perception of illness severity, reported signs and symptoms, the time interval between illness onset and the study, and access to disease surveillance information, among others.

### Data analysis and statistical methods

We performed complex sample survey data analysis to account for the differential probabilities of selecting respondents in the study due to the complex sampling approach. We incorporated survey weights in the complex survey data analysis and computed weighted estimates, standard errors, and confidence intervals. Univariate data analysis was conducted to describe respondents’ characteristics and health-seeking patterns. We obtained weighted summary statistics and proportions with confidence intervals for continuous and categorical variables respectively. We conducted weighted crude multinomial logistic regression analysis to examine the unadjusted association between each explanatory variable and the outcome, with ‘facility care’ as the outcome reference category. Adopting a backward stepwise regression approach, we fitted weighted multivariable multinomial logistic regression models to adjust for confounders and identify factors associated with health-seeking patterns. We obtained weighted adjusted odds ratios with confidence intervals for the explanatory variables. Akaike Information Criterion (AIC) was employed to evaluate the parsimony of the multivariable model candidates with penalization for complexity. We selected the model with the lowest AIC value as the most parsimonious for our data. Multicollinearity among the explanatory variables in the multivariable models was assessed using variance inflation factor (VIF). The VIF values for all the variables were less than 5, indicating low multicollinearity among these variables [30, 31]. Data were analyzed using R statistical and computing software version R-4.2.2 [32].

### Reporting

We reported this study based on the Guidelines for Reporting Observational Studies in keeping with the Strengthening the Reporting of Observational Studies in Epidemiology (STROBE) Statement [33].

## Results

A total of 2,373 IDPs participated in the study. All selected households were surveyed with no refusals. Similarly, all sampled participants responded to interviews directly or through their caregivers (if < 18 years). The weighted median age of the respondents was 30 years (95% CI: 30–32 years), with an interquartile range of 15 years. Among the respondents, 38.6% (95% CI: 35.3–42.0) were 18 to 29 years old, while 33.2% (95% CI: 30.1–36.0) were between the ages of 30 and 39 years. Furthermore, 78.1% (95% CI: 75.3–81.0) were females, 81.0% (95% CI: 78.4–84.0) had no formal education, 64.4% (95% CI: 61.2–68.0) were unemployed, and 62.6% (95% CI: 59.3–66.0) had lived in IDPs camps for more than five years (Table 1).

**Table 1:** Demographic characteristics of respondents

Characteristics (N = 2373)		Number of respondents	Weighted percent (95% CI)
**Age group (years)**			
	18–29	796	38.6 (35.3–42.0)
	30–39	763	33.2 (30.1–36.0)
	40–49	436	16.4 (14.0–19.0)
	≥ 50	378	11.8 (9.6–14.0)
**Sex**			
	Male	799	21.9 (19.2–25.0)
	Female	1574	78.1 (75.3–81.0)
**Highest Education level attained**			
	None	1797	81.0 (78.4–84.0)
	Primary	346	13.7 (11.4–16.0)
	Post primary (secondary, tertiary)	230	5.3 (3.8–7.0)
**Marital Status**			
	Never married	234	10.5 (8.4–12.0)
	Presently married	1805	73.8 (70.9–77.0)
	Widowed	200	9.0 (7.1–11.0)
	Others*	134	6.7 (5.0–10.0)
**Religion**			
	Islam	2215	99.4 (99.2–100.0)
	Christianity	158	0.6 (0.3–1.0)
**Occupation**			
	Unemployed	1056	64.4 (61.2–68.0)
	Farmers	855	13.3 (11.2–15.0)
	Traders/Business	263	14.3 (12.0–17.0)
	Artisan (skilled labourer)	109	4.3 (2.9–6.0)
	Students	51	3.0 (1.9–4.0)
	Others†	39	0.7 (0.2–1.0)
**Monthly household income**			
	< 13,300 NGN^¶^	1698	80.7 (78.1–83.0)
	≥ 13,300 NGN	675	19.3 (16.7–22.0)
**Duration of residence in IDPs camp**			
	≤ 5 years	812	37.4 (34.2–41.0)
	> 5 years	1561	62.6 (59.3–66.0)

* Separated, divorced, cohabiting

^†^ Civil servants, unskilled laborers, drivers

^¶^ Nigerian Naira (13,300 NGN = 30 United States Dollars)

Of the 2,373 respondents, 95.9% (95% CI: 94.5–97.0) reported illness onset within one month prior to the study, while 73.9% (95% CI: 70.9–77.0) reported illness onset within two weeks. Table 2 shows the common reported signs and symptoms of most recent illness; 79.3% of the respondents (95% CI: 76.5–82.0) reported fever, 51.2% (95% CI: 47.9–55.0) reported cough, while 47.1% (95% CI: 43.7–50.0) reported diarrhea.

**Table 2:** Reported signs and symptoms of most recent illnesses

Reported signs and symptoms	Number of respondents	Weighted percent (95% CI)
Fever	1904	79.3 (76.5–82.0)
Cough	1409	51.2 (47.9–55.0)
Diarrhoea	1171	47.1 (43.7–50.0)
Vomiting	904	36.2 (33.0–39.0)
Headache	634	33.1 (29.9–36.0)
Body pain	339	12.5 (10.3–15.0)
Body rash	197	8.3 (6.4–10.0)
Neck stiffness	30	1.0 (0.4–2.0)

Among the respondents, 75.7% (95% CI: 72.9–78.6) sought ‘facility care’, 11.1% (95% CI: 9.1–13.1) sought ‘non-facility care’, while 13.2% (95% CI: 10.9–15.4) practiced ‘home care/no care’. The patterns of health-seeking by respondents’ characteristics are presented in supplementary file 1. Among the respondents that ‘sought facility care’ a large proportion were females (78.5%), had no formal education (81.1%), married (73.1%), and resided in official camps (98.9%).

Table 3 presents weighted crude and adjusted odds ratios from multinomial logistic regression analysis of factors associated with health-seeking patterns. Respondents who perceived that illness was severe compared to those who did not (Adjusted Odds Ratio (AOR) = 0.15, [95% CI: 0.08–0.30]), respondents who resided in official camps compared to unofficial camps (AOR = 0.26, [95% CI: 0.17–0.39]) and respondents who lived in households with mother as decision maker on health seeking (AOR = 0.33, [95% CI: 0.18–0.60]) were significantly less likely to seek ‘non-facility care’ rather than ‘facility care’. Moreover, respondents who had lived in IDPs camps for more than 5 years (AOR = 3.47, [95% CI: 2.00–6.01]), and respondents who perceived IDP camps were secured (AOR = 2.31, [95% CI: 1.25–4.29]) were significantly more likely to seek ‘non-facility care’ compared to ‘facility care’.

**Table 3:** Results of weighted multinomial logistic regression analysis of factors associated with health-seeking pattern

Participants’ characteristics		Non-facility care versus facility care ^ψ^				Home care/no care versus facility care ^ψ^		
		COR^†^ (95% CI)		AOR^‡^ (95% CI)		COR^†^ (95% CI)		AOR^‡^ (95% CI)
**Perception of illness severity**								
	Not severe (reference)							
	Severe	0.14 (0.08–0.26)		0.15 (0.08–0.30)*		0.65 (0.42–0.99)		0.72 (0.46–1.13)
**Status of IDPs camp**								
	Informal (reference)							
	Formal	0.15 (0.11–0.19)		0.26 (0.17–0.39)*		0.32 (0.24–0.42)		0.58 (0.36–0.92)*
**Duration of residence in IDPs camp**								
	≤ 5 years (reference)							
	> 5 years	4.76 (2.85–7.94)		3.47 (2.00–6.01)*		2.83 (1.77–4.51)		2.41 (1.47–3.95)*
**Decision maker on health seeking**								
	Self (reference)							
	Father	0.70 (0.37–1.34)		0.62 (0.30–1.28)		1.47 (0.83–2.59)		1.32 (0.72–2.41)
	Mother	0.28 (0.16–0.47)		0.33 (0.18–0.60)*		0.71 (0.45–1.11)		0.64 (0.39–1.04)
	Others (uncles, aunts, in-laws)	1.74 (0.79–3.83)		1.37 (0.59–3.16)		1.35 (0.52–3.51)		1.42 (0.53–3.83)
**Distance to nearest health facility**								
	< 2 km (reference)							
	≥ 2 km	0.35 (0.21–0.59)		0.53 (0.26–1.07)		3.1 (1.98–4.88)		2.72 (1.53–4.84)*
**Monthly household income**								
	< 13,300 NGN^¶^ (reference)							
	≥ 13,300 NGN	5.86 (3.78–9.09)		3.38 (2.07–5.51)*		0.93 (0.54–1.62)		0.90 (0.50–1.64)
**Received disease surveillance information**								
	No (reference)							
	Yes	1.76 (0.98–3.15)		1.89 (0.94–3.78)		0.33 (0.22–0.50)		0.42 (0.26–0.67)*
**Perception of IDPs camp security**								
	Insecure (reference)							
	Secure	3.45 (2.02–5.88)		2.31 (1.25–4.29)*		0.46 (0.31–0.69)		0.85 (0.49–1.48)
**Highest formal educational level attained**								
	None (reference)							
	Primary	2.04 (1.21–3.43)		1.04 (0.57–1.92)		0.63 (0.32–1.24)		0.76 (0.38–1.54)
	Post Primary (Secondary and tertiary)	1.32 (0.61–2.85)		0.73 (0.28–1.88)		0.24 (0.07–0.85)		0.30 (0.08–1.18)
**Age group (years)**								
	18–29 (reference)							
	30–39	0.95 (0.59–1.54)		0.89 (0.50–1.57)		1.03 (0.63–1.68)		0.97 (0.56–1.69)
	40–49	0.66 (0.35–1.27)		0.61 (0.26–1.40)		1.03 (0.56–1.87)		0.99 (0.50–1.96)
	≥ 50	1.04 (0.53–2.05)		0.65 (0.29–1.45)		1.89 (1.05–3.41)		1.51 (0.73–3.11)
**Marital status**								
	Never married (reference)							
	Presently married	1.12 (0.57–2.21)		1.35 (0.65–2.81)		1.35 (0.67–2.73)		0.78 (0.32–1.87)
	Widowed	0.77 (0.28–2.09)		0.93 (0.32–2.69)		1.59 (0.64–3.92)		1.10 (0.38–3.20)
	Others (separated, divorced, cohabiting)	0.79 (0.27–2.32)		1.18 (0.37–3.79)		1.07 (0.37–3.10)		0.70 (0.21–2.39)
**Sex**								
	Male (reference)							
	Female	0.59 (0.38–0.92)		1.43 (0.86–2.40)		1.42 (0.85–2.37)		1.60 (0.89–2.85)
**Religion**								
	Islam (reference)							
	Christianity	6.20 (2.43–15.83)		13.69 (0.70–267.16)		1.5 (0.56–4.05)		2.16 (0.62–7.51)
**Age of household member with most recent illness**								
	< 18 years (reference)							
	≥ 18 years	2.55 (1.66–3.94)		1.19 (0.64–2.21)		1.30 (0.87–1.94)		1.57 (0.80–3.08)

^ψ^ Reference category

^†^ Weighted Crude Odds Ratio

^‡^ Weighted Adjusted Odds Ratio

^¶^ Nigerian Naira (13,300 NGN = 30 United States Dollars)

* Statistically significant at *P* < 0.05

Furthermore, respondents who resided in official camps compared to unofficial camps (AOR = 0.58, [95% CI: 0.36–0.92]), and respondents who received disease surveillance information (AOR = 0.42, [95% CI: 0.26–0.67]) were significantly less likely to practice ‘home care/no care’ rather than seek ‘facility care’. Additionally, respondents who had lived in IDPs camps for more than 5 years (AOR = 2.41, [95% CI: 1.47–3.95]), and respondents who resided 2 km or farther from the nearest health facility (AOR = 2.72, [95% CI: 1.53–4.84]) were significantly more likely to practice ‘home care/no care’ rather than seek ‘facility care’.

## Discussion

This multi-states, multi-camps research provides considerable insight into the patterns and factors associated with health-seeking among IDPs in complex humanitarian emergency, as part of a broader effort to enhance disease surveillance and response in Northeast Nigeria. The study also contributes to the scarce literature on this subject. We found that the patterns of health-seeking among this population were heterogeneous. Although most of the respondents sought care from health facility during illness, a number of them also sought ‘non-facility care’ and practiced ‘home care/no care’. Nearly all of them reported illness onset within a month prior to this study. Fever, cough, and diarrhea were the most reported signs and symptoms. Perception of illness severity, status of IDPs camps, decision maker on health-seeking, duration of residence in IDPs camps, and access to disease surveillance information were significantly associated with health-seeking patterns among this population.

The diverse patterns of health-seeking among IDPs in the current study draws attention to the practice of medical pluralism in this context. Medical pluralism refers to the utilization of different treatment approaches and options to maintain health or treat illness [34]. Approximately 25% of respondents did not seek care at the health facility during illness episodes, similar to the findings of Marwat et al. in Pakistan [35]. Such respondents either sought care from non-facility service providers or practiced ‘home care/no care’. Although facility-based surveillance is recognized as an important surveillance approach in humanitarian contexts [12, 36], our result indicates that this surveillance approach alone may not be adequate to meet the surveillance needs of crisis-affected population. This finding suggests that relying solely on facility-based surveillance could lead to delayed or missed detection of diseases and other conditions, precluding the opportunity for timely and appropriate interventions. Considering the necessity to strengthen disease surveillance and response in this context, our study provides emerging evidence to expand the surveillance network beyond health facilities to include PMV, traditional healers, religious homes, and other non-facility service providers.

Nearly all respondents reported illness onset within a month prior to the study, similar to findings of previous research [24]. This result reflects the high prevalence of risk factors for disease transmission in humanitarian contexts and indicates that IDPs are burdened by enormous health problems, corroborating existing literature [37, 38]. The enormity of diseases and other health conditions among the IDPs could overwhelm the existing surveillance system, particularly in view of the fragile health system, further exposing the vulnerability of this population. This narrative justifies the need to strengthen disease surveillance and response in this context, bolstering the views of other authors [39, 40]. Furthermore, for programme planning purposes, the huge health problems among the IDPs could be underestimated by data generated solely via facility-based surveillance. Results from our study suggest that over-reliance on data from this surveillance approach could misguide local health authorities and undermine effective programme planning and implementation in this context.

The majority of respondents in this study reported fever, cough, and diarrhea as the most common symptoms of their recent illnesses. These features are suggestive of common epidemic-prone diseases such as measles, cholera, and cerebrospinal meningitis in the study area [41]. Moreover, these symptoms could also indicate diseases of public health importance, notably malaria, pneumonia, and diarrhea which are among the leading causes of deaths in under-5 children globally [42, 43]. In certain instances, some of these diseases could present in milder forms as low severity illnesses and subsequently managed at home or by non-facility service providers. Because such mild ailments might not necessitate care seeking at health facility, they are likely to be detected late or missed completely by facility-based surveillance. In line with our earlier submission, this description reflects the need to broaden the surveillance network and intensify active case search in households and among non-facility service providers, particularly in epidemic situations to enable prompt detection of such cases for appropriate intervention, in accordance with recommended global practices [14].

Our study revealed that respondents who perceived that illness was severe were less likely to seek ‘non-facility care’ rather than ‘facility care’. This finding is consistent with results obtained in previous studies conducted in Ethiopia, Burkina Faso, and the United Kingdom [19, 20, 44]. According to the conceptual framework of the Health Belief Model, the Extended Parallel Process Model, and other models of health behaviour, perceived illness severity is among the key constructs predicting health-related behaviours, especially uptake of health care services [45, 46]. Essentially, individuals strive to overcome geographical, contextual, and socio-cultural barriers to seek facility care for perceived severe ailments due to fear of complications and deaths. Regardless of their heterogenous health-seeking patterns, our result suggests that IDPs recognize and trust health facilities to manage severe illnesses. Thus, to promote care-seeking at health facility and optimize facility-based surveillance in this setting, local health authorities should leverage this trust and scale up risk communication activities to shape disease risk perception and trigger a higher level of perceived illness severity among this population.

Findings from our study indicated that respondents who resided in official (formal) IDPs camps, compared to unofficial camps, were less likely to seek ‘non-facility care’ and less likely to practice ‘home care/no care’ rather than ‘facility care’. This result could be attributed to the differences in the attributes of official and unofficial camps. Unlike unofficial IDPs camps, official camps are formally designated and well-recognized by the Government. These camps enjoy the presence and support of appropriate Government authorities and Non-Governmental Organizations (NGOs). In addition to treatment of common ailments, IDPs in official camps also, benefit from other health services, including family planning, antenatal care, management of malnutrition, and routine immunization services offered by the Government and NGOs in these camps at a free or heavily subsidized cost. We believe that this health care support is likely to attract IDPs residing in official camps and influence their decision to seek care at health facility. While the ongoing efforts by the Government to return IDPs to their homes are acknowledged and commendable, this finding highlights the need for local health authorities to prioritize IDPs currently residing in unofficial camps for health care delivery and disease surveillance interventions including community health outreaches and active case search activities.

We found that duration of residence in IDPs camps was significantly associated with health-seeking patterns. IDPs who had resided in the camps for a prolonged duration were more likely to seek ‘non-facility care’ and practice ‘home care/no care’ rather than ‘facility care’. During the early phase of internal displacement, IDPs often explore available social, health, and economic support to promote their welfare and facilitate their adaptation to the new environment. In this regard, IDPs are more likely to patronize health facilities during the acute phase of displacement to seek care and leverage the initial Government-coordinated, facility-based health interventions. However, with prolonged displacement, particularly in the face of dwindling humanitarian assistance, many IDPs are deprived of opportunities to support themselves and meet most basic necessities, including the uptake of essential health services at the facilities [47]. Additionally, prolonged displacement typically disrupts progress in health care, education, and other key sectors fundamentals to health and socio-economic development [47, 48]. Furthermore, with prolonged stay in the camps, a number of IDPs, especially women and female children, might have experienced physical attacks, sexual assault, and other forms of violence [49]. The psychological trauma from such experiences could negatively influence these IDPs and possibly deter them from utilizing institutionalized establishments, including health facilities.

In this study, health-seeking decisions taken by mothers were less likely to favor ‘non-facility care’ compared to ‘facility care’. In many settings, particularly in rural Africa, mothers typically frequent health facilities alone or with their children to access maternal and child health care services. Such repeated visitations and experiences presumably enhance their familiarity with health facility service delivery and build their confidence to utilize health facility during illness episodes. Moreover, in northeast Nigeria, where this study was implemented, mothers are generally, regarded as the primary caregivers of the children. With this role, they are able to promptly recognize danger signs of severe illness and likely to exhibit a higher level of perceived illness severity, which as earlier reported, was negatively associated with seeking ‘non-facility care’. The foregoing interpretation could also, partly explain why women, especially mothers, are mostly engaged as voluntary community mobilizers by Government and NGOs in northern Nigeria to educate, sensitize and mobilize community members for immunization activities, disease surveillance, and other health interventions in the region [50].

This research targeted displaced population within the milieu of armed conflict-induced humanitarian crisis. In spite of the challenging terrain, we leveraged available data sources, program reports, and micro-planning data from various organizations to optimize the validity of our study. For instance, the IOM DTM assessment reports that guided the selection of our study camps are considered among the most comprehensive and reliable displacement reports globally [7]. Nevertheless, we recognize certain limitations in this study. Firstly, data on health-seeking patterns and several explanatory variables were retrospective, self-reported, and not verified. Therefore, the potential for recall bias and misinformation cannot be ruled out. Secondly, we were limited by security concerns in this region and thus, selected study IDPs camps from locations perceived to be relatively safe at the time of the study. Presumably, health-seeking patterns among IDPs residing in camps located in active armed-conflict zones might be different from the patterns in secure areas with potential implications for disease surveillance and response. Thirdly, the selection of our study IDPs camps was largely guided by data; nonetheless, we acknowledge that the purposive selection of these camps might have possibly introduced selection bias, particularly because IDPs camps in this region enjoy differing health and humanitarian support. Furthermore, our assessment of illness severity was based on respondents’ perception rather than the actual measurement of disease severity. Findings from this study are generalizable to displaced populations residing within IDPs camps in this region but may not be applicable to IDPs already integrated and residing in host communities. Because of their special status, specific rights, and international protection, findings from this study may not apply to refugees.

## Conclusions

This study permits a better understanding of health-seeking patterns among IDPs in northeast Nigeria and provides valuable epidemiological evidence to inform appropriate strategies to enhance disease surveillance and response in this context. Although the IDPs predominantly sought care from facility service providers, a number of them sought ‘non-facility care’ and practiced ‘home care/no care’ during illness episodes. Perception of illness severity, status of IDPs camps, household health-seeking decision maker, and duration of residence in IDPs camps were significantly associated with health-seeking patterns. To enhance prompt disease detection for timely and appropriate intervention in this context, our study cautions against over-reliance on facility-based surveillance and recommends the expansion of disease surveillance network beyond health facilities to include chemist, PMVs, traditional healers, religious and faith-based homes, and other non-facility care providers. Leveraging women influencers, authorities should scale up health education and risk communication activities to shape disease risk perception and promote care-seeking during illness episodes. Surveillance actors should intensify active case search in households and among non-facility service providers to enable prompt disease detection and timely response. Interventions to enhance disease surveillance in this context should prioritize IDPs residing in unofficial camps and those with prolonged displacement.

### Electronic supplementary material

Below is the link to the electronic supplementary material.

Supplementary Material 1

## Data Availability

This study was conducted in the context of complex humanitarian emergency triggered by armed insurgency against the Nigerian Government following the activities of Non-State Armed Groups. Given the enormous security risk and concerns, the data collected and analyzed from the study are not publicly available. However, the data are available from the corresponding author on reasonable request.

## Footnotes

1 Household listing data are generated through walk-through enumeration activity – an essential component of the immunization program’s micro-planning exercise.

## Abbreviations

AIC: Akaike information criterion
AOR: Adjusted odds ratio
CHE: Complex Humanitarian Emergency
CI: Confidence interval
COVID-19: Coronavirus disease
DTM: Displacement Tracking Matrix
IDPs: Internally displaced persons
IOM: International Organization for Migration
LGA: Local Government Area
NGOs: Non-Government Organizations
NHREC: National Health Research Ethics Committee of Nigeria
ODK: Open data kit
PMVs: Patent medicine vendors
SSA: Sub-Saharan Africa
STROBE: Strengthening the reporting of observation studies in epidemiology
UN OCHA: United Nations Office for the Coordination of Humanitarian Affairs
VIF: Variance inflation factor
WASH: Water, sanitation, and hygiene
WHO: World Health Organization
