# Supplementary material for: Factors associated with health-seeking patterns among internally displaced persons in complex humanitarian emergency, Northeast Nigeria: a cross-sectional study
Source: Confl Health. 2023 Nov 8;17:54. doi: 10.1186/s13031-023-00552-7 (PMC10630990; doi:10.1186/s13031-023-00552-7)
Supplement: Supplementary file 1 — Supplementary Material 1 [file 13031_2023_552_MOESM1_ESM.docx]

| **Supplementary file 1: Patterns of health-seeking among internally displaced persons by key characteristics** | | | | | | | | | |
| --- | --- | --- | --- | --- | --- | --- | --- | --- | --- |
| **Respondents' characteristics** | | **Facility care** | |  | **Non-facility care** | |  | **Home care/no care** | |
|  |  | **Number of respondents**  **(N = 1385)** | **Weighted percent (95% CI)** |  | **Number of respondents**  **(N = 614)** | **Weighted percent (95% CI)** |  | **Number of respondents**  **(N = 374)** | **Weighted percent (95% CI)** |
| **Age group (years)** | | | | | | | | | |
|  | 18 - 29 | 477 | 38.8 (35.0 - 42.5) |  | 206 | 41.7 (32.1 - 51.2) |  | 113 | 34.9 (26.2 - 43.7) |
|  | 30 - 39 | 449 | 33.4 (29.8 - 37.1) |  | 215 | 34.3 (25.2 - 43.3) |  | 99 | 31.0 (22.5 - 39.5) |
|  | 40 - 49 | 263 | 17.2 (14.3 - 20.1) |  | 104 | 12.2 (6.1 - 18.3) |  | 69 | 15.9 (9.2 - 22.6) |
|  | ≥ 50 | 196 | 10.6 (8.3 - 13.0) |  | 89 | 11.8 (5.7 - 18.0) |  | 93 | 18.2 (11.2 - 25.2) |
| **Sex** | |  |  |  |  |  |  |  |  |
|  | Male | 423 | 21.5 (18.3 - 24.7) |  | 245 | 31.7 (22.9 - 40.6) |  | 131 | 16.2 (9.7 - 22.7) |
|  | Female | 962 | 78.5 (75.3 - 81.7) |  | 369 | 68.3 (59.4 - 77.1) |  | 243 | 83.8 (77.3 - 90.3) |
| **Highest formal educational level attained** | |  |  |  |  |  |  |  |  |
|  | None | 1084 | 81.1 (78.1 - 84.1) |  | 416 | 70.3 (61.5 - 79.0) |  | 297 | 89.3 (83.9 - 94.8) |
|  | Primary | 188 | 13.1 (10.5 - 15.7) |  | 109 | 23.1 (14.9 - 31.4) |  | 49 | 9.2 (4.0 - 14.4) |
|  | Post primary (secondary, tertiary) | 113 | 5.8 (4.0 - 7.6) |  | 89 | 6.6 (2.3 - 10.8) |  | 28 | 1.5 (0.3 - 3.3) |
| **Marital status** | |  |  |  |  |  |  |  |  |
|  | Never married | 119 | 10.8 (8.4 - 13.3) |  | 67 | 10.3 (4.5 - 16.1) |  | 48 | 8.2 (3.3 - 13.2) |
|  | Presently married | 1082 | 73.1 (70.0 - 76.5) |  | 460 | 77.8 (68.8 - 86.7) |  | 263 | 75.0 (67.1 - 82.9) |
|  | Widowed | 105 | 9.0 (6.8 - 11.3) |  | 48 | 6.6 (1.9 - 11.3) |  | 47 | 11.0 (5.3 - 16.6) |
|  | Others* | 79 | 7.1 (5.1 - 9.1) |  | 39 | 5.3 (1.1 - 9.5) |  | 16 | 5.8 (1.5 - 10.1) |
| **Religion** | |  |  |  |  |  |  |  |  |
|  | Islam | 1333 | 99.6 (99.3 - 99.9) |  | 532 | 97.8 (97.2 - 98.4) |  | 350 | 99.5 (99.3 - 99.7) |
|  | Christianity | 52 | 0.4 (0.04 - 0.7) |  | 82 | 2.2 (1.6 - 2.8) |  | 24 | 0.5 (0.3 - 0.8) |
| **Monthly household income** | |  |  |  |  |  |  |  |  |
|  | < 13,300 NGN^¶^ | 1081 | 84.6 (81.8 - 87.4) |  | 337 | 48.4 (38.8 - 58.0) |  | 280 | 85.5 (79.2 - 91.8) |
|  | ≥ 13,300 NGN | 304 | 15.4 (12.6 - 18.2) |  | 277 | 51.6 (42.0 - 61.2) |  | 94 | 14.5 (8.2 - 20.8) |
| **Decision maker on health seeking** | |  |  |  |  |  |  |  |  |
|  | Self (respondent) | 674 | 37.6 (33.8 - 41.3) |  | 388 | 57.8 (48.3 - 67.4) |  | 207 | 40.4 (31.5 - 49.4) |
|  | Father | 210 | 11.8 (9.3 - 14.3) |  | 81 | 12.8 (6.4 - 19.2) |  | 70 | 18.6 (11.5 - 25.8) |
|  | Mother | 414 | 47.2 (43.3 - 51.1) |  | 71 | 20.1 (12.2 - 28.0) |  | 72 | 35.9 (27.0 - 44.8) |
|  | Others (uncles, aunts, in-laws) | 87 | 3.5 (2.1 - 4.9) |  | 74 | 9.3 (3.8 - 14.7) |  | 25 | 5.1 (1.1 - 9.0) |
| **Perception of illness severity** | |  |  |  |  |  |  |  |  |
|  | Not severe | 834 | 57.3 (53.5 - 61.2) |  | 495 | 90.5 (85.4 - 95.7) |  | 293 | 67.5 (58.9 - 76.2) |
|  | Severe | 551 | 42.7 (38.8 - 46.5) |  | 119 | 9.5 (4.3 - 14.6) |  | 81 | 32.5 (23.8 - 41.1) |
| **Status of IDPs camp** | |  |  |  |  |  |  |  |  |
|  | Informal | 321 | 1.1 (1.0 - 1.2) |  | 303 | 6.8 (5.5 - 8.2) |  | 172 | 3.3 (2.6 - 4.0) |
|  | Formal | 1064 | 98.9 (98.8 - 99.0) |  | 311 | 93.2 (91.8 - 94.5) |  | 202 | 96.7 (96.0 - 97.4) |
| **Duration of residence in the camp** | |  |  |  |  |  |  |  |  |
|  | ≤ 5 years | 479 | 43.6 (39.8 - 47.5) |  | 214 | 14.0 (8.1 - 19.8) |  | 119 | 21.5 (14.0 - 28.9) |
|  | > 5 years | 906 | 56.4 (52.5 - 60.2) |  | 400 | 86.0 (80.2 - 91.9) |  | 255 | 78.5 (71.1 - 85.9) |
| **Distance to the nearest health facility** | |  |  |  |  |  |  |  |  |
|  | < 2km | 1167 | 86.3 (83.7 - 89.0) |  | 433 | 94.7 (92.4 - 97.0) |  | 265 | 67.0 (58.4 - 75.7) |
|  | ≥ 2km | 218 | 13.7 (11.0 - 16.3) |  | 181 | 5.3 (3.0 - 7.6) |  | 109 | 33.0 (24.3 - 41.6) |
| **Received disease surveillance information** | |  |  |  |  |  |  |  |  |
|  | No | 285 | 20.4 (17.2 - 23.5) |  | 126 | 12.7 (6.6 - 18.8) |  | 196 | 43.8 (34.7 - 52.9) |
|  | Yes | 1100 | 79.6 (76.5 - 82.8) |  | 488 | 87.3 (81.2 - 93.4) |  | 178 | 56.2 (47.1 - 65.3) |
| **Perception of IDPs camp security** | |  |  |  |  |  |  |  |  |
|  | Insecure | 437 | 26.5 (23.1 - 29.9) |  | 206 | 9.5 (5.1 - 13.8) |  | 108 | 43.9 (34.8 - 53.1) |
|  | Secure | 948 | 73.5 (70.1 - 76.9) |  | 408 | 90.5 (86.2 - 94.9) |  | 266 | 56.1 (46.9 - 65.2) |

* Separated, divorced, cohabiting

^¶^ Nigerian Nigeria (13,300 NGN = 30 US Dollars)
